# Does Timing Matter? Associations Between Intimate Partner Violence Across the Early Life Course and Internalizing and Externalizing Behavior in Children

**DOI:** 10.1177/08862605231174505

**Published:** 2023-05-24

**Authors:** Katrina M. Moss, Deborah Loxton, Gita D. Mishra

**Affiliations:** 1 The University of Queensland, Herston, Australia; 2 University of Newcastle, NSW, Australia

**Keywords:** intimate partner violence, internalizing behavior, externalizing behavior, life course, child, Australian Longitudinal Study on Women’s Health

## Abstract

The association between exposure to intimate partner violence (IPV) and child behavior problems is well established. However, questions remain about whether the timing during the child’s early life course matters. We used a structured life course approach to investigate associations between the timing of IPV and children’s internalizing and externalizing behaviors. Participants were from the Australian Longitudinal Study on Women’s Health (ALSWH), a national, randomly sampled community-based study that has surveyed women every 3 years since 1996. For this study, mothers born 1973 to 1978 (*N* = 2,163) provided data on their three youngest children under 13 years (*N* = 3,697, 48.5% female) as part of the Mothers and their Children’s Health (MatCH) study in 2016/2017. Mothers indicated IPV in ALSWH using the Community Composite Abuse Scale in early (*M* = 0.99 years, *SD* = 0.88 years) and middle childhood (*M* = 3.98 years, *SD* = 0.92 years), and before birth (preconception). Mothers rated child internalizing and externalizing behavior in MatCH (child age: *M* = 8.15 years, *SD* = 2.37 years) using the Strengths and Difficulties Questionnaire. We tested critical period, sensitive period, and accumulation hypotheses by comparing the fit of nested linear regression models (separately for girls and boys). Mothers were predominantly Caucasian (>90%) and university educated (65.5%), and 41.7% reported financial stress. Most children were not exposed to IPV (68.1%). Of those who were, 55.2% were exposed at one time, 28.7% at two times, and 16.1% at all three. Accumulation was the best model for externalizing in boys and girls and for internalizing in girls. A critical period in middle childhood was identified for internalizing in boys. Overall, the duration of exposure was more important than the timing. This suggests early detection is essential in mitigating the impact of IPV on children, with particular attention needed for boys exposed to IPV in middle childhood.

The association between exposure to intimate partner violence (IPV) and behavior problems in children is well established (Fogarty et al., 2019; Fong et al., 2019; Kitzmann et al., 2003; McTavish et al., 2016; Wolfe et al., 2003). IPV is a pattern of behavior that can include physical, sexual, psychological and/or emotional violence, or threats of violence (Fong et al., 2019; Hegarty et al., 2005; Loxton et al., 2013). IPV has high prevalence, with 30% to 50% of women reporting IPV in their lifetime (Fogarty et al., 2019; Wood & Sommers, 2011). Approximately 25% of children are exposed to IPV (Fogarty et al., 2019), which can include witnessing or being aware of IPV directed to one or both parents (Fong et al., 2019). IPV can also adversely affect the mental health and parenting styles of caregivers (FitzPatrick et al., 2020; Skinner et al., 2019) and can cluster with other adverse exposures such as poverty (Skinner et al., 2019), although IPV crosses all ethnic and socioeconomic boundaries (Skinner et al., 2019; Wood & Sommers, 2011). Up to two-thirds of children exposed to IPV will have poorer outcomes than their peers, such as lower cognitive functioning, social and behavioral problems, sleep disturbances, and mental health issues such as anxiety and post-traumatic stress (Fogarty et al., 2019).

Internalizing (e.g., anxiety, fear, depression, withdrawal, and somatic symptoms) and externalizing behaviors (e.g., aggression, hyperactivity, conduct, impulsivity, substance use, and attention problems) are frequently studied in relation to IPV (Achenbach et al., 2016; Fogarty et al., 2019; Vu et al., 2016; Wolfe et al., 2003). Although some children will adapt (Fogarty et al., 2019), children exposed to IPV are more likely to experience internalizing and/or externalizing problems (Vu et al., 2016; Wolfe et al., 2003). This can set in motion an adverse developmental pathway. For example, externalizing problems may appear as attachment problems in infancy, behavior problems in preschool, aggression and poor social skills at school age, and delinquency and crime in adolescence and adulthood (Artz et al., 2014).

Several meta-analyses have found that evidence of sex differences in the context of IPV is mixed. Vu et al. (2016) found that the child’s sex did not influence the correlation between IPV and internalizing or externalizing behavior. Similarly, Wolfe et al. (2003) found that effect sizes were similar for boys and girls. The child’s sex as a moderator was not significant in Kitzmann et al. (2003), but evidence was mixed in Fong et al. (2019). Wood and Sommers (2011) concluded that boys and girls may react differently to witnessing IPV, but this may be influenced by the sex of the perpetrator and whether outcomes are measured in the short or long term. There are also sex differences in emotional and behavioral problems, with boys generally showing more externalizing and girls more internalizing (Fong et al., 2019; Hawes & Dadds, 2004).

While the association between IPV and some child outcomes appears clear, questions remain about whether the timing of exposure is important. This is critical information because it can inform the optimal scheduling of screening and interventions. However, there are inconsistent findings on the role of child age (Fong et al., 2019), perhaps because many studies are cross-sectional (Vu et al., 2016). This prevents conclusions about which age groups may be more vulnerable, with unknown age at first exposure and confounding between timing and the amount of exposure identified as key methodological weaknesses (Fong et al., 2019). These can be overcome by using a structured life course approach to rigorously test critical period, sensitive period, and accumulation hypotheses (Mishra et al., 2009). A critical period of development suggests that poorer outcomes result from exposure at one specific part of the life course, and the exposure outside of this window of time does not convey excess risk (Mishra et al., 2015). Critical periods are more commonly found with biological exposures, and fetal programing is one example of this (Mishra et al., 2015). A sensitive period of development suggests that poorer outcomes can be associated with exposure during several parts of the life course, but the effect will be stronger for one particular time, and this is more commonly found for behavioral development (Mishra et al., 2015). These timing-based hypotheses posit that adverse events can be particularly potent when exposure occurs during times of high developmental plasticity, where interruptions can disrupt the development of biological subsystems that regulate stress and emotions (Dunn et al., 2018; Mishra et al., 2015). In contrast, the accumulation hypothesis suggests a dose–response or cumulative association, where each additional exposure is associated with additional risk, regardless of when it occurs (Mishra et al., 2015).

Versions of these structured life course models have been tested in the context of child abuse but not IPV. For example, studies from the Avon Longitudinal Study of Parents and Children (ALSPAC) have identified accumulation as the best model for the association between child abuse from age group 1 to 9 years and social cognition at age group 7 to 14 years (Crawford et al., 2020). Accumulation and recency were identified as the best models for the association between child abuse in the early life course and child emotional and behavioral problems at 8 years of age (Dunn et al., 2018). However, in a subsample from ALSPAC, Dunn et al. (2019) found that sensitive period rather than accumulation was the best explanation for the association between exposure to adversity and DNA methylation at 7 years, with 0 to 3 years identified as a sensitive period. Some IPV-focused studies have explored timing, but not with the same level of rigor. For example, Silva et al. (2019) found stronger associations with child emotional and behavioral problems when IPV was first reported between 1 and 2 years of age, but that associations were also significant for IPV prenatally, in the first postnatal year and from 3 to 6 years of age. This would suggest a sensitive period; however, models were not tested against each other or against accumulation hypotheses. Although there is overlap between child abuse and IPV (Brown et al., 2021), they are not the same and we cannot assume that results will generalize.

Our study used data from a national longitudinal community-based sample to investigate the role of timing in the association between IPV and children’s internalizing and externalizing problems. Specifically, we wanted to understand whether critical period, sensitive period, or accumulation hypotheses provided the best explanation of the association between IPV exposure at three time points (preconception, in early childhood, and/or in middle childhood), and internalizing and externalizing behavior in later childhood.

## Method

### Participants

Participants in this study were from the Australian Longitudinal Study on Women’s Health (ALSWH; alswh.org.au). Women from three age-based cohorts (1921–1926, 1946–1951, and 1973–1978) were randomly selected from Australia’s universal health insurance system in 1996 and were representative of the Australian population at the time of recruitment (Lee et al., 2005). However, due to attrition over time and changing population demographics, the current ALSWH sample is under-representative on cultural diversity and over-represents women with a university education (Dobson et al., 2015). Maternal data in this study came from women in the cohort born in 1973 to 1978 (*N* = 14,247). They have completed up to seven surveys between 1996 and 2015, at approximately 3-yearly intervals. Child data in this study came from the Mothers and their Children’s Health (MatCH) study, which is part of ALSWH. For MatCH, ALSWH mothers (*N* = 3,039) provided cross-sectional information on their three youngest children aged under 13 years (*N* = 5,780) in 2016/2017 (Mishra et al., 2018). In other words, this is a maternal cohort rather than a birth cohort: mothers are of a similar age, but child ages range from 0 to 12 years, and births could have occurred at any time from 2004.

The children included in the current study were: (a) children whose first survey after birth was ALSWH survey 4, 5, or 6; (b) children whose mother had provided information on IPV in three consecutive ALSWH surveys with the first occurring before the child’s birth (preconception) and the following two occurring after the child’s birth; and (c) children who had valid internalizing and externalizing scores on the Strengths and Difficulties Questionnaire (SDQ) (*N* = 3,697 children from *N* = 2,163 mothers).

Ethical approval was obtained from both the University of Queensland and the University of Newcastle, and mothers provided informed written consent for themselves and their children.

### Measures

#### Intimate Partner Violence

Mothers were first asked about IPV in ALSWH survey 3 (2003, women aged 25–30 years). A summary question asked women whether they had experienced, in the last 3 years: physical abuse (e.g., pushed, grabbed, kicked, etc.); severe physical violence (e.g., beaten up, thrown, choked, etc.); emotional abuse (e.g., threatened, called names, humiliated, bullied, etc.); sexual abuse (e.g., rape or attempted rape, sexual assault, forced to engage in unwanted sexual practices); and/or harassment (e.g., stalking, loitering, offensive telephone calls, etc.). From survey 4 (2006, women aged 28 to 33 years) onward, women were administered the Community Composite Abuse Scale (CCAS; Loxton et al., 2013), which includes most items from the Composite Abuse Scale (Hegarty et al., 2005), but instead of four questions on sexual abuse, it included a summary question deemed less confronting for a community-based sample (i.e., “Forced me to take part in unwanted sexual activity”). It is a valid, reliable, and acceptable measure of IPV in a community sample (Loxton et al., 2013).

For the current study, women were categorized as experiencing IPV if they reported at least one behavior from the CCAS that had occurred within the last 12 months. To explore IPV across the child’s early life course, ratings of maternal IPV were taken from three consecutive ALSWH surveys approximately 3 years apart: (a) the ALSWH survey before the child’s birth, which we have labeled “preconception”; (b) the first ALSWH survey after the child’s birth—children were aged 0.99 years on average (*SD* = 0.88 years, range = 0–3 years) at this survey, and we have labeled this time point “early childhood”; and (c) the second ALSWH survey after the child’s birth—children were aged 3.98 years on average (*SD* = 0.92 years, range = 2–7 years) and we have labeled this time point “middle childhood.”

#### Internalizing and Externalizing Behavior

In 2016/2017, mothers rated children’s internalizing and externalizing behavior using the SDQ (Goodman, 1997) as part of MatCH. The SDQ is a valid and reliable measure of emotional and behavioral problems in Australian children (Hawes & Dadds, 2004) and is commonly used in epidemiological studies (Dunn et al., 2018). Questions are rated on a 3-point scale (not true, somewhat true, and certainly true). Internalizing includes five items each on emotional symptoms and peer relationship problems, and externalizing includes five items each on conduct problems and hyperactivity/inattention. Cronbach’s alpha for total difficulties in our study (.82) was the same as Australian norms (.82) (Hawes & Dadds, 2004).

#### Covariates

Maternal covariates were taken from the ALSWH survey closest to MatCH (i.e., most proximal to the child outcome measure). These were the mother’s ability to manage on income (dichotomized as 0 = easy/not too bad; 1 = difficult sometimes/always/impossible) and maternal education (dichotomized as 0 = less than university; 1 = university or higher university), based on the association between socioeconomic status, IPV and child’s emotional and behavioral problems (Howell et al., 2016; McTavish et al., 2016; Skinner et al., 2019). Child covariates were age at MatCH (in years) and whether the child had any siblings in MatCH (0 = No; 1 = Yes). Maternal mental health and parenting are both associated with child outcomes in the context of IPV (Fogarty et al., 2019). However, these are on the causal pathway—they can both result from experiencing IPV (Fong et al., 2019). We did not include them as covariates as adjusting for intermediate variables can bias the estimates (Schisterman et al., 2009; van Zwieten et al., 2022).

### Statistical Analysis

Initial descriptive statistics were run in SAS (version 9.4, SAS Institute Inc., Cary, NC). IPV timing groups were constructed based on whether or not IPV was reported at three times during the child’s early life course: preconception, early childhood (first ALSWH survey after the child’s birth), and middle childhood (second ALSWH study after the child’s birth). This led to eight mutually exclusive IPV groups: (a) none (0 0 0); (b) preconception only (1 0 0); (c) early childhood only (0 1 0); (d) middle childhood only (0 0 1); (e) preconception and early childhood (1 1 0); (f) early and middle childhood (0 1 1); (g) preconception and middle childhood (1 0 1); and (h) all three times (1 1 1).

The structured life course analysis (Mishra et al., 2009; Mishra et al., 2013) was conducted in MPLUS (version 8, Muthen & Muthen, Los Angeles, CA). This approach compares the fit of a series of nested models to a reference (saturated) model. Linear regression models were fitted using maximum likelihood estimation. Models were run without covariates first (unadjusted), and then with covariates added (adjusted). Models were multilevel to account for children nested within mothers, were run separately for boys and girls, and used robust standard errors (MLR estimator – Maximum Likelihood with Robust standard errors) to account for non-normality in SDQ scores. The reference/saturated model estimated the coefficient for every combination of timing by including a dummy variable for each of the seven IPV groups, with “none” used as the reference category. The critical period hypothesis assumed an effect would only be present for one time period and was tested by estimating the coefficient for IPV during each period of the life course in three separate nested models: preconception only (1 0 0) versus all other children, early childhood only (0 1 0) versus all other children, and middle childhood only (0 0 1) versus all other children. The sensitive period hypothesis assumed associations would be present for more than one period of the life course, but these would not be equal in strength. It was tested by estimating the coefficients for all three periods in one nested model by simultaneously entering IPV preconception only (1 0 0), early childhood only (0 1 0), and middle childhood only (0 0 1). The accumulation hypothesis assumed the coefficients for each time were equivalent (i.e., that timing did not matter) and that the duration of exposure was most important. It was tested by summing the number of times the mother reported IPV (0–3 times). The sum was entered as a continuous variable (0–3) in one nested model to investigate whether duration of exposure was associated with child behavior, and as a categorical variable (0, 1, 2, and 3) in another nested model to investigate whether the association was linear (e.g., whether the coefficient for IPV exposure at one time period was similar in size to the coefficient for exposure at two or three time periods or whether there was a dose–response type effect).

The fit of the nested critical period, sensitive period, and accumulation models was compared with the saturated model using likelihood difference tests (accounting for the MLR estimation by using a scaling correction factor) (Muthen & Muthen) and *p*-values based on chi-square distributions. Compared with the saturated model, the model with the lowest values of Akaike’s information criterion (AIC) and *p*-values closest to 1 was selected as the best-fitting model. After using model fit indices to determine the best-fitting model for the data, interpretation of the best-fitting model was based on model coefficients and confidence intervals.

#### Sensitivity Analysis

As ALSWH is a maternal rather than a child cohort, there was variation in child ages at the first two surveys after the child’s birth: from 0 to 3 years at the first post-birth survey and 2 to 7 years at the second survey (Supplemental Table 1). To investigate potential confounding due to overlapping age categories, we ran a second set of analyses that only included children aged 0 to 2 at the first post-birth survey and aged 3 to 7 at the second post-birth survey.

## Results

### Sample

The sample for the current study included 3,697 children from2,163 mothers. Mothers on average were aged 31.99 years (*SD* = 2.57 years, range = 25–38 years) at the child’s birth. In terms of diversity, mothers were predominantly Caucasian (>90%) Australian-born women. Approximately two in five lived outside major cities, one in three did not have a university education, and approximately 13% reported substantial difficulty managing on their available income. Children on average were aged 8.15 years (*SD* = 2.37 years, and range = 3–12 years) at the time of MatCH (when outcomes were measured). Almost half (48.5%) were female. Table 1 presents demographic characteristics for boys and girls. There were no major differences, except fewer boys had siblings in MatCH. Table 1 also presents information on the outcome variables and shows that more boys scored in the raised/high category on the SDQ, and this was mainly due to higher externalizing scores.

**Table 1.** Demographic Characteristics, IPV Exposure, and Outcome Variables for Boys and Girls.

Variable	Boys *N* = 1,903 (51.5%)	Girls *N* = 1,794 (48.5%)	*p*-Value
Demographic characteristics			
Maternal age at child’s birth (*M*, *SD*)	31.99 (2.59)	32.00 (2.55)	.952
Maternal ability to manage income (*N*, %)^a^			
Easy	367 (19.3)	329 (18.3)	.677
Not too bad	734 (38.6)	725 (40.4)	
Difficult sometimes	542 (28.5)	506 (28.2)	
Difficult always/impossible	260 (13.7)	234 (13.0)	
Maternal educational level (*N*, %)^a^			
≤Year 12	218 (11.5)	240 (13.4)	.149
Trade/Diploma	415 (21.8)	402 (22.4)	
University	1,270 (66.7)	1,152 (64.2)	
Area of residence (*N*, %)^a^			
Major city	1,078 (56.7)	1,023 (57.0)	.733
Inner region	535 (28.1)	486 (27.1)	
Outer region/remote/very remote	290 (15.2)	285 (15.9)	
Had siblings in MatCH (*N*, %)^b^			
Yes	1,682 (88.4)	1,537 (85.7)	.014
No	221 (11.6)	257 (14.3)	
Age at early childhood survey (*M*, *SD*)^c^	0.99 (0.88)	0.99 (0.88)	.908
Age at middle childhood survey (*M*, *SD*)^d^	3.98 (0.92)	3.98 (0.91)	.914
Age at outcome measurement (*M*, *SD*)^e^	8.13 (2.34)	8.16 (2.39)	.733
Exposure to maternal IPV			
Timing across life course (*N*, %)			
None	1,290 (67.8)	1,228 (68.5)	.529
Preconception only	144 (7.6)	135 (7.5)	
Early childhood only	96 (5.0)	73 (4.1)	
Middle childhood only	111 (5.8)	92 (5.1)	
Preconception and early childhood	55 (2.9)	48 (2.7)	
Early and middle childhood	87 (4.6)	80 (4.5)	
Preconception and middle childhood	29 (1.5)	39 (2.2)	
All three times	91 (4.9)	99 (5.5)	
No. times exposed to IPV (*N*, %)			
0 time point	1,290 (67.8)	1,228 (68.5)	.441
1 time point	351 (18.4)	300 (16.7)	
2 time points	171 (9.0)	167 (9.3)	
3 time points	91 (4.8)	99 (5.5)	
Outcome variables			
SDQ total difficulties score^b^			
Average	1,622 (85.2)	1,614 (90.0)	<.001
Raised/high	281 (14.8)	180 (10.0)	
SDQ internalizing score (*M*, *SD*)^b^	2.65 (2.84)	2.62 (2.72)	.768
SDQ externalizing score (*M*, *SD*)^b^	5.10 (3.65)	3.88 (3.23)	<.001

*Note. p*-Values are from chi-square tests of independence for categorical variables and independent sample *t*-tests for continuous variables. ALSWH = Australian Longitudinal Study on Women’s Health; MatCH = Mothers and their Children’s Health Study; IPV = intimate partner violence; SDQ = Strengths and Difficulties Questionnaire.

a Taken from the ALSWH survey closest to MatCH (i.e., most proximal to outcome measurement).

b Taken from the MatCH survey.

c First ALSWH survey completed after the child’s birth (early childhood, children aged 0–3 years).

d Second ALSWH survey after the child’s birth (middle childhood, children aged 2–6 years).

e MatCH survey in 2016/2017 (children aged 4–12 years).

### IPV Across the Early Life Course

Approximately two-thirds of children (68.1%) did not experience IPV at any time point. Of those who did, just over half (*n* = 651, 55.2%) experienced IPV at one time point (preconception *n* = 279 [42.9%]; early childhood *n* = 169 [26.0%]; and middle childhood *n* = 203 [31.2]). Just over one-quarter (*n* = 338, 28.7%) experienced IPV at two time points (preconception and early childhood *n* = 103 [30.5%]; early and middle childhood *n* = 167 (49.4%); and preconception and middle childhood *n* = 68 [20.1%]). Fewer children (*n* = 190, 16.1%) experienced IPV at all three time points. There were no differences between boys and girls (Table 1).

### IPV and Child Externalizing Problems

#### Descriptive Statistics

**Figure 1.**
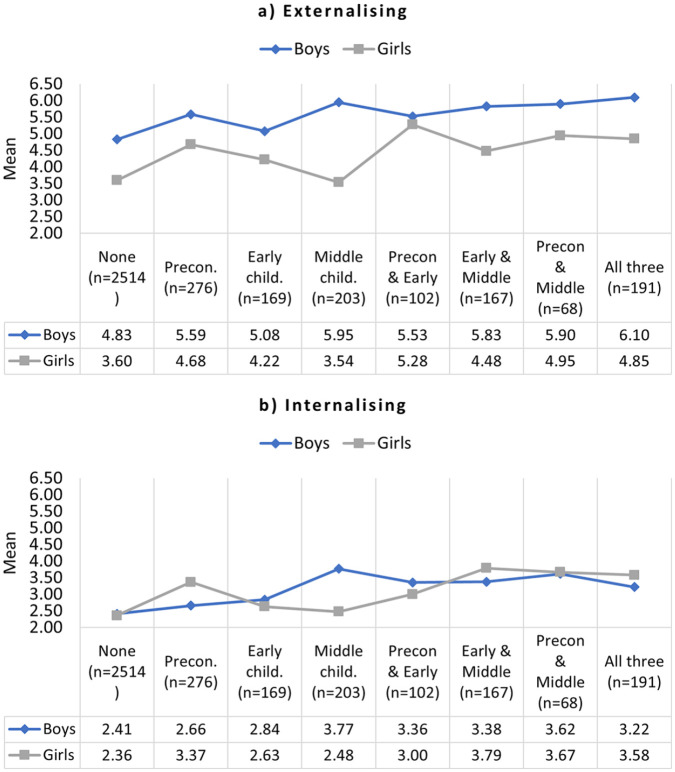
Mean externalizing (a) and internalizing (b) scores for boys and girls, by timing of IPV exposure across the early life course (*N* = 3,697). *Note*. IPV = intimate partner violence.

Mean externalizing scores for boys and girls with different life course exposures to IPV can be seen in Figure 1a. Scores were generally higher for boys, but trajectories were parallel, with two exceptions. There was a larger difference between boys’ and girls’ scores for children exposed to IPV in middle childhood only, with scores for boys rising and scores for girls dropping. There was a smaller difference between boys’ and girls’ scores for children exposed to IPV at both preconception and early childhood, with scores for girls rising.

**Figure 2.**
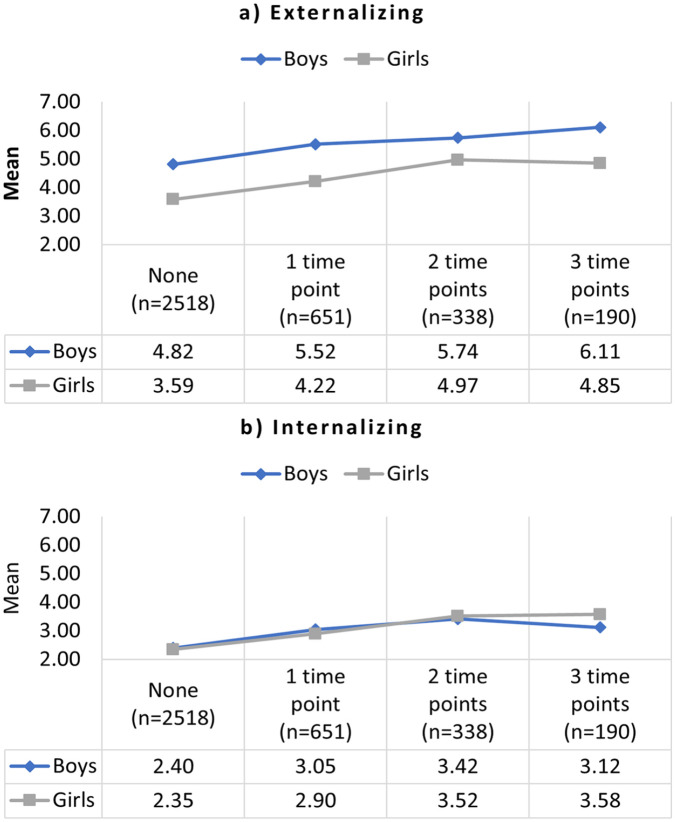
Mean externalizing (a) and internalizing (b) scores for boys and girls, by number of time periods exposed to IPV (*N* = 3,697). *Note*. IPV = intimate partner violence.

Mean externalizing scores by number of time periods exposed to IPV can be seen in Figure 2a. For both boys and girls, the relationship was generally linear: the more time points children were exposed to IPV, the higher the mean scores.

#### Life Course Modeling: Model Fit

Based on AIC and *p*-values, accumulation (continuous) was the best-fitting model of the adjusted association between IPV exposure and externalizing problems in both boys and girls (Table 2; see Supplemental Table 2 for model fit for unadjusted models). Compared to the saturated model, fit for the critical period and sensitive period models was poor. However, the continuous accumulation model had the highest *p*-value and lowest AIC value, suggesting fit was not significantly worse than the saturated/reference model, indicating this more parsimonious model was the best fit for the data.

**Table 2.** Model Fit for Structured Life Course Models of the Adjusted Association Between IPV and Child Externalizing and Internalizing Behavior in Boys and Girls.

Models	Log likelihood	*p*-Value	AIC	Scaling correction	Free parameters
Externalizing					
Boys (adjusted)					
Saturated^a^	−5,076.56	NA	10,183.12	1.1163	15
Critical: preconception^b^	−5,085.28	.016	10,188.55	1.1176	9
Critical: early childhood^b^	−5,086.12	.012	10,190.24	1.0855	9
Critical: middle childhood^b^	−5,084.09	.035	10,186.18	1.1197	9
Sensitive period^c^	−5,082.95	.027	10,187.91	1.0993	11
Accumulation: continuous^d^	**−5,078.99**	**.639**	**10,175.98**	**1.1043**	**9**
Accumulation: categorical^e^	−5,078.25	.546	10,178.50	1.1213	11
Girls (adjusted)					
Saturated^a^	−4,511.43	NA	9,052.86	1.2026	15
Critical: preconception^b^	−4,524.73	.002	9,067.45	1.1429	9
Critical: early childhood^b^	−4,526.70	.001	9,071.40	1.0909	9
Critical: middle childhood^b^	−4,527.16	<.001	9,072.33	1.1428	9
Sensitive period^c^	−4,523.04	.004	9,068.08	1.0919	11
Accumulation: Continuous^d^	**−4,514.89**	**.488**	**9,047.78**	**1.1571**	**9**
Accumulation: categorical^e^	−4,514.32	.332	9,050.63	1.1816	11
Internalizing					
Boys (adjusted)					
Saturated^a^	−4,599.42	NA	9,228.83	1.2973	15
Critical: preconception^b^	−4,612.92	.003	9,243.84	1.2687	9
Critical: early childhood^b^	−4,613.05	.002	9,244.11	1.2778	9
Critical: middle childhood^b^	**−4,605.16**	**.163**	**9,228.31**	**1.3293**	**9**
Sensitive period^c^	−4,605.08	.094	9,232.17	1.2501	11
Accumulation: continuous^d^	−4,607.69	.045	9,233.37	1.3067	9
Accumulation: categorical^e^	−4,605.67	.051	9,233.34	1.2883	11
Girls (adjusted)					
Saturated^a^	−4,245.83	NA	8,521.66	1.2823	15
Critical: preconception^b^	−4,262.17	<.001	8,542.35	1.4025	9
Critical: early childhood^b^	−4,267.24	<.001	8,552.49	1.3335	9
Critical: middle childhood^b^	−4,267.06	<.001	8,552.12	1.3410	9
Sensitive period^c^	−4,261.90	<.001	8,545.80	1.2930	11
Accumulation: continuous^d^	**−4,251.86**	**.103**	**8,521.72**	**1.3753**	**9**
Accumulation: categorical^e^	−4,251.45	.026	8,524.90	1.3778	11

*Note*. AIC = Akaike’s information criterion; IPV = intimate partner violence. Bolded lines represent the best-fitting model.

a The saturated model includes dummy variables for all possible combinations of the timing of IPV (“none” is the reference category).

b The three critical period models include a dummy variable for IPV: (a) preconception only; (b) early childhood only; or (c) middle childhood only.

c The sensitive period model includes dummy variables for IPV preconception only, early childhood only, and middle childhood only.

d The continuous accumulation model uses a single variable with the values of 0 to 3.

e The categorical accumulation model uses dummy variables for IPV once, twice or three times (0 times is the reference category).

#### Life Course Modeling: Interpretation

The continuous accumulation model shows that, compared to no exposure to IPV, exposure at any one time was associated with higher externalizing problems, although associations were larger when IPV was reported at two or three times during the early life course (Supplemental Figure 1 for boys and Supplemental Figure 2 for girls; see Supplemental Table 3 for regression coefficients for unadjusted models). The regression coefficient for the continuous accumulation model was similar between boys (coefficient = 0.39; 95% CI [0.18, 0.59]) and girls (coefficient = 0.45; [0.26, 0.64])

### IPV and Child Internalizing Problems

#### Descriptive Statistics

Mean internalizing scores for boys and girls with different life course exposures to IPV can be seen in Figure 1b. Scores were generally similar for boys and girls with two exceptions. In children exposed to IPV in preconception only, scores were higher for girls. In children exposed to IPV in middle childhood only, scores were higher for boys.

Mean internalizing scores by number of time periods exposed to IPV can be seen in Figure 2b. For girls, the relationship was generally linear: the more time points children were exposed to IPV, the higher the mean scores. For boys, the relationship was linear for 0 to 2 time periods of IPV exposure, but mean scores dropped for boys exposed to IPV at three time periods.

#### Life Course Modeling: Model Fit

For girls, the continuous accumulation model had the highest *p*-value and lowest AIC value, suggesting it was the best-fitting model of the adjusted association between IPV exposure and internalizing problems (Table 2, see Supplemental Table 2 for model fit for unadjusted models). Critical and sensitive period models were not supported.

#### Life Course Modeling: Interpretation

For girls, the continuous accumulation model shows that, compared to no exposure to IPV, exposure at any one time was associated with higher internalizing problems, although associations were larger when IPV was reported at two or three parts of the early life course (Supplemental Figure 3; see Supplemental Table 4 for regression coefficients for unadjusted models).

#### Life Course Modeling: Model Fit

For boys, middle childhood was identified as a critical period for internalizing problems. The critical period model for middle childhood had the lowest AIC values and the highest *p*-values, suggesting this more parsimonious model was a better fit for the data than the saturated model.

#### Life Course Modeling: Interpretation

For boys, the confidence intervals for preconception and early childhood critical models indicate statistical nonsignificance, and the coefficients are also quite small (Supplemental Figure 4; see Supplemental Table 4 for regression coefficients for unadjusted models). In contrast, the coefficient for the middle childhood critical model is large and statistically significant. This suggests that IPV was not associated with internalizing problems unless it occurred during middle childhood.

### Sensitivity Analysis

Models were re-run on a smaller sample of children (*n* = 3,467) where age ranges for the first and second post-birth ALSWH surveys did not overlap. The pattern of results was the same, with accumulation the best model for externalizing in boys and girls, and for internalizing in girls. As above, a critical period in middle childhood was identified for internalizing in boys (Supplemental Tables 5 and 6).

## Discussion

This study used data from a national prospective longitudinal study to test the association between the timing of maternal IPV during the child’s early life course and subsequent internalizing and externalizing problems. Comparing IPV reported in preconception, early childhood, and middle childhood, it found that timing was not important for externalizing problems in either boys or girls—the duration of exposure to IPV was most strongly related to child outcomes. This same result held for internalizing problems in girls, with more time periods of exposure associated with higher internalizing behavior. However, middle childhood was identified as a critical period of exposure in boys, with higher internalizing problems for boys only evident when they were exposed to IPV in middle childhood. Overall, the results suggest early detection is essential for IPV, and boys exposed to IPV in middle childhood may be particularly vulnerable in terms of internalizing problems.

The accumulation model suggests that exposure to IPV at any stage of the life course is associated with poorer outcomes for children, but that exposure over a longer period of time is worse—in other words, a cumulative effect (Mishra et al., 2015). Child maltreatment studies have supported accumulation models for child behavior (Dunn et al., 2018) and social cognitive skills (Crawford et al., 2020). In contrast, Yoon (2022) found sensitive periods explained the association between child adversity from 5 to 16 years and externalizing problems at 17 years; however, this may reflect the children’s age at measurement, as externalizing problems can increase during adolescence. Life course models have not been tested within the IPV literature, but a comparison can be drawn to the “sleeper effect” proposed by (Holmes, 2013), where effects are small or not evident early on but increase over time. Under more rigorous testing, this may have been a cumulative effect (Vu et al., 2016). Meta-analyses have found similar effects for IPV at different parts of the life course (Kitzmann et al., 2003; Wolfe et al., 2003), or that results are mixed (Fong et al., 2019). One meta-analysis found that child age at time of IPV assessment was associated with externalizing problems, but that age at time of outcome assessment was associated with internalizing problems (Vu et al., 2016). The high number of cross-sectional studies make it difficult to disentangle age effects. More large-scale prospective longitudinal studies are needed to rigorously investigate the effects of developmental timing, and this should be a priority for future research (Woodard & Pollak, 2020; Wood & Sommers, 2011).

The critical period model suggests that exposure has to occur within a particular limited time window to influence development, and if it occurs outside this window, then there is no elevated risk (Mishra et al., 2015). During critical periods, biobehavioral systems are more receptive to environmental influences (Woodard & Pollak, 2020). In our study, internalizing problems were only identified for boys who were exposed to IPV in middle childhood (2–6 years), and not early childhood (0–3 years) or preconception. Exposure to IPV can impair emotion regulation via stress-related cortisol disruptions and lack of healthy modeling of emotion regulation processes (Howell et al., 2016), in particular how to understand and interpret conflict (Carlson et al., 2019). This can make it more difficult for children to establish healthy relationships, and caregiver modeling is particularly influential for preschool-aged children (3–6 years) (Howell et al., 2016). Despite this clear theoretical justification, the findings of the current study need to be replicated in longitudinal studies that measure IPV and child outcomes across the early life course. A rigorous, replicated understanding of the effects of timing can inform screening and intervention, and potentially increase the efficiency of public spending in the area of IPV, and this is an important area for future research.

This study had several strengths. It was longitudinal, which allowed testing of the life course models as we had maternal reports of IPV across the child’s early life course. It had a large sample size, which enabled separate analysis of data for boys and girls. Our sample was national- and community-based, rather than shelter- or clinic-based, which strengthens the generalization of results. Finally, we used a broad definition of IPV that included harassment and physical, emotional, and sexual abuse, enabling a comprehensive understanding of the negative impact of IPV (Vu et al., 2016). However, there were some important limitations. The first relates to the limited diversity in our sample in terms of maternal age, education, and cultural background. As the IPV measure was only introduced in survey 3, mothers were aged 25 to 38 years at the child’s birth, and so results may not generalize to younger mothers. Similarly, mothers with a university level education were over-represented. However, financial stress was reported by 42% of the sample, including mothers with a university education, and IPV does cross class boundaries (Wood & Sommers, 2011). Additionally, our cohort was representative of the Australian population at recruitment, but this occurred prior to the migration boom and our primarily Caucasian sample is not representative of Australia’s current cultural diversity. All of these points should be considered when generalizing the findings from this study and should be addressed in future research investigating life course exposures to IPV. The second limitation was that we did not have measures of IPV perpetrated by the mother, and so our study may underestimate the child’s exposure to IPV, although a meta-analysis of longitudinal studies on IPV and child behavior found no difference in correlations when only partner IPV was included or when both maternal and partner IPV was included (Vu et al., 2016). Similarly, our question captured IPV in the last 12 months, but survey intervals were 3 years, so this may also have resulted in an underestimate of IPV. The final limitation is that our measure of IPV did not capture the severity or frequency of IPV, and we acknowledge that there is likely to be heterogeneity of IPV experience. We believe this reflects community-based samples, although we would suggest that future research explores IPV frequency and severity when testing life course models.

This study affirms the association between IPV and child internalizing and externalizing problems and contributes novel evidence on the role of timing. Longer exposure was associated with poorer outcomes for children, underscoring the need for early identification for parents experiencing IPV. With regard to internalizing problems, middle childhood appears to be a particularly vulnerable time for boys, and this may need to be a focus for screening and intervention. Overall though, findings suggest that intervention efforts do not need to be directed to particular parts of the life course, but instead need to focus on detecting and intervening as early as possible to reduce children’s exposure to IPV.

## Supplemental Material

sj-docx-1-jiv-10.1177_08862605231174505 – Supplemental material for Does Timing Matter? Associations Between Intimate Partner Violence Across the Early Life Course and Internalizing and Externalizing Behavior in Children Click here for additional data file. Supplemental material, sj-docx-1-jiv-10.1177_08862605231174505 for Does Timing Matter? Associations Between Intimate Partner Violence Across the Early Life Course and Internalizing and Externalizing Behavior in Children by Katrina M. Moss, Deborah Loxton and Gita D. Mishra in Journal of Interpersonal Violence
