# Supplementary material for: Does Timing Matter? Associations Between Intimate Partner Violence Across the Early Life Course and Internalizing and Externalizing Behavior in Children
Source: J Interpers Violence. 2023 May 24;38(19-20):10566–87. doi: 10.1177/08862605231174505 (PMC10466944; doi:10.1177/08862605231174505)

**Appendix A. Supplementary materials**

Supplementary Table 1

*Child ages at the first and second post-birth ALSWH surveys*

| Child age (years) | 1^st^ post-birth  survey  N (%) | 2^nd^ post-birth  survey  N (%) |
| --- | --- | --- |
| 0 | 1311 (35.5) | - |
| 1 | 1247 (33.7) | - |
| 2 | 994 (26.9) | 85 (2.3) |
| 3 | 145 (3.9) | 1195 (32.3) |
| 4 | - | 1256 (34.0) |
| 5 | - | 1026 (27.8) |
| 6 | - | 132 (3.6) |
| 7 | - | 3 (0.1) |

Supplementary Table 2

*Model fit for structured life course models of the unadjusted association between IPV and child externalizing and internalizing behavior in boys and girls.*

|  | Log  likelihood | P  value | AIC | Scaling  correct | Free  Params |
| --- | --- | --- | --- | --- | --- |
| **Externalizing** |  |  |  |  |  |
| **Boys (unadjusted)** |  |  |  |  |  |
| Saturated^a^ | -5122.81 | NA | 10265.62 | 1.1612 | 10 |
| Critical: pre-conception^b^ | -5132.69 | .007 | 10273.39 | 1.2187 | 4 |
| Critical: early childhood^b^ | -5133.74 | .005 | 10275.48 | 1.1326 | 4 |
| Critical: middle childhood^b^ | -5131.58 | .016 | 10271.16 | 1.2170 | 4 |
| Sensitive period^c^ | -5130.18 | .014 | 10272.36 | 1.1491 | 6 |
| Accumulation: Continuous^d^ | **-5125.07** | **.685** | **10258.15** | **1.1826** | **4** |
| Accumulation: Categorical^e^ | -5124.48 | .560 | 10260.96 | 1.1922 | 6 |
| Saturated^a^ | -5133.81 | .007 | 10273.62 | 1.2313 | 3 |
| **Girls (unadjusted)** |  |  |  |  |  |
| Saturated^a^ | -4596.84 | NA | 9213.68 | 1.3178 | 10 |
| Critical: pre-conception^b^ | -4611.85 | .001 | 9231.70 | 1.3032 | 4 |
| Critical: early childhood^b^ | -4613.64 | <.001 | 9235.28 | 1.2054 | 4 |
| Critical: middle childhood^b^ | -4613.51 | <.001 | 9235.02 | 1.2894 | 4 |
| Sensitive period^c^ | -4610.92 | .001 | 9233.83 | 1.1820 | 6 |
| Accumulation: Continuous^d^ | **-4600.07** | **.554** | **9208.14** | **1.3268** | **4** |
| Accumulation: Categorical^e^ | -4599.70 | .363 | 9211.40 | 1.3156 | 6 |
| Empty model | -4614.07 | <.001 | 9234.15 | 1.3516 | 3 |
| **Internalizing** |  |  |  |  |  |
| **Boys (unadjusted)** |  |  |  |  |  |
| Saturated^a^ | -4629.69 | NA | 9279.374 | 1.4182 | 10 |
| Critical: pre-conception^b^ | -4648.22 | <.001 | 9304.45 | 1.5408 | 4 |
| Critical: early childhood^b^ | -4648.21 | <.001 | 9304.42 | 1.5622 | 4 |
| Critical: middle childhood^b^ | -4639.82 | .012 | 9287.64 | 1.6899 | 4 |
| Sensitive period^c^ | -4639.56 | .007 | 9291.11 | 1.4313 | 6 |
| Accumulation: Continuous^d^ | -4638.25 | .039 | 9284.49 | 1.6112 | 4 |
| Accumulation: Categorical^e^ | -4635.28 | .080 | 9282.56 | 1.4691 | 6 |
| Empty model | -4648.22 | <.001 | 9302.45 | 1.7607 | 3 |
| **Girls (unadjusted)** |  |  |  |  |  |
| Saturated^a^ | -4256.60 | NA | 8533.19 | 1.4396 | 10 |
| Critical: pre-conception^b^ | -4277.69 | <.001 | 8563.39 | 1.9084 | 4 |
| Critical: early childhood^b^ | -4283.57 | <.001 | 8575.14 | 1.7562 | 4 |
| Critical: middle childhood^b^ | -4283.60 | <.001 | 8575.21 | 1.7589 | 4 |
| Sensitive period^c^ | -4277.58 | <.001 | 8567.15 | 1.5325 | 6 |
| Accumulation: Continuous^d^ | **-4262.57** | **.112** | **8533.14** | **1.8629** | **4** |
| Accumulation: Categorical^e^ | -4262.08 | .032 | 8536.16 | 1.7119 | 6 |
| Empty model | -4283.77 | <.001 | 8573.53 | 2.0850 | 3 |

^a^The saturated model includes dummy variables for all possible combinations of the timing of IPV (“none” is the reference category).

^b^The three critical period models include a dummy variable for IPV: 1) pre-conception only; 2) early childhood only; or 3) middle childhood only.

^c^The sensitive period model includes dummy variables for IPV pre-conception only, early childhood only and middle childhood only.

^d^The continuous accumulation model uses a single variable with the values of 0 to 3.

^e^The categorical accumulation model uses dummy variables for IPV once, twice or three times (0 times is the reference category)

Supplementary Table 3

*Unadjusted regression coefficients for life course models of the association between intimate partner violence reported pre-conception, early and middle childhood, and externalizing behavior in boys and girls.*

|  | | Boys | Girls |
| --- | --- | --- | --- |
| Timing of Intimate Partner Violence | | Regression coefficient  (95% CI) | Regression coefficient  (95% CI) |
| **Saturated model^a^** | |  |  |
| 0 0 0 | None | 0 (Reference) | 0 (Reference) |
| 1 0 0 | Pre-conception only | 0.69 (0.01, 1.36) | 0.84 (0.24, 1.44) |
| 0 1 0 | Early childhood only | 0.20 (-0.49, 0.88) | 0.66 (-0.01, 1.32) |
| 0 0 1 | Middle childhood only | 0.99 (0.22, 1.75) | -0.05 (-0.76, 0.66) |
| 1 1 0 | Pre-concept. & early child. | 0.47 (-0.58, 1.52) | 1.37 (0.22, 2.58) |
| 1 0 1 | Early & middle childhood | 0.96 (0.05, 1.88) | 0.84 (-0.03, 1.71) |
| 0 1 1 | Pre-concept. & middle child. | 0.95 (-0.38, 2.29) | 1.39 (0.21, 2.57) |
| 1 1 1 | All 3 times | 1.22 (0.37, 2.07) | 1.27 (0.51, 2.03) |
| **Critical period models^b^** | |  |  |
| 1 0 0 | Pre-conception only | 0 (Reference) | 0 (Reference) |
|  |  | 0.46 (-0.19, 1.11) | 0.61 (0.02, 1.20) |
| 0 1 0 | Early childhood only | 0 (Reference) | 0 (Reference) |
|  |  | -0.14 (-0.82, 0.54) | 0.36 (-0.29, 1.00) |
| 0 0 1 | Middle childhood only | 0 (Reference) | 0 (Reference) |
|  |  | 0.75 (-0.01, 1.52) | -0.38 (-1.08, 0.33) |
| **Sensitive period model^c^** | |  |  |
| 1 0 0 | Pre-conception only | 0.52 (-0.15, 1.18) | 0.61 (0.02, 1.21) |
| 0 1 0 | Early childhood only | 0.02 (-0.67, 0.70) | 0.39 (-0.27, 1.04) |
| 0 0 1 | Middle childhood only | 0.80 (0.04, 1.57) | -0.28 (-0.99, 0.43) |
| **Accumulation model^d^ (continuous)** | |  |  |
|  | No. times IPV | 0.44 (0.23, 0.64) | 0.48 (0.29, 0.68) |
| **Accumulation model^e^ (categorical)** | |  |  |
|  | 0 | 0 (Reference) | 0 (Reference) |
|  | 1 | 0.65 (0.19, 1.11) | 0.52 (0.10, 0.95) |
|  | 2 | 0.80 (0.15, 1.46) | 1.12 (0.50, 1.75) |
|  | 3 | 1.22 (0.37, 2.07) | 1.27 (0.51, 2.03) |

^a^The saturated model includes dummy variables for all possible combinations of the timing of IPV (“none” is the reference category).

^b^The three critical period models include a dummy variable for IPV: 1) pre-conception only; 2) early childhood only; or 3) middle childhood only.

^c^The sensitive period model includes dummy variables for IPV pre-conception only, early childhood only and middle childhood only.

^d^The continuous accumulation model uses a single variable with the values of 0 to 3.

^e^The categorical accumulation model uses dummy variables for IPV once, twice or three times (0 times is the reference category)

Supplementary Table 4

*Unadjusted regression coefficients for life course models of the association between intimate partner violence reported pre-conception, early and middle childhood, and internalizing behavior in boys and girls.*

|  | | Boys | Girls |
| --- | --- | --- | --- |
| Timing of Intimate Partner Violence | | Regression coefficient  (95% CI) | Regression coefficient  (95% CI) |
| **Saturated model^a^** | |  |  |
| 0 0 0 | None | 0 (Reference) | 0 (Reference) |
| 1 0 0 | Pre-conception only | 0.25 (-0.20, 0.70) | 1.02 (0.44, 1.59) |
| 0 1 0 | Early childhood only | 0.37 (-0.19, 0.93) | 0.17 (-0.37, 0.70) |
| 0 0 1 | Middle childhood only | 1.33 (0.66, 2.00) | 0.14 (-0.37, 0.65) |
| 1 1 0 | Pre-concept. & early child. | 0.75 (-0.23, 1.72) | 0.64 (-0.09, 1.36) |
| 1 0 1 | Early & middle childhood | 1.12 (0.44, 1.80) | 1.34 (0.63, 2.05) |
| 0 1 1 | Pre-concept. & middle child. | 1.00 (-0.20, 2.21) | 1.29 (0.37, 2.22) |
| 1 1 1 | All 3 times | 0.63 (-0.04, 1.30) | 1.32 (0.64, 1.99) |
| **Critical period models^b^** | |  |  |
| 1 0 0 | Pre-conception only | 0 (Reference) | 0 (Reference) |
|  |  | -0.01 (-0.44, 0.43) | 0.82 (0.25, 1.39) |
| 0 1 0 | Early childhood only | 0 (Reference) | 0 (Reference) |
|  |  | 0.05 (-0.51, 0.61) | -0.20 (-0.73, 0.34) |
| 0 0 1 | Middle childhood only | 0 (Reference) | 0 (Reference) |
|  |  | 1.13 (0.46, 1.79) | -0.17 (-0.67, 0.33) |
| **Sensitive period model^c^** | |  |  |
| 1 0 0 | Pre-conception only | 0.09 (-0.35, 0.54) | 0.81 (0.23, 1.38) |
| 0 1 0 | Early childhood only | 0.19 (-0.36, 0.75) | -0.12 (-0.67, 0.42) |
| 0 0 1 | Middle childhood only | 1.16 (0.49, 1.82) | -0.10 (-0.60, 0.40) |
| **Accumulation model^d^ (continuous)** | |  |  |
|  | No. times IPV | 0.36 (0.19, 0.54) | 0.50 (0.34, 0.67) |
| **Accumulation model^e^ (categorical)** | |  |  |
|  | 0 | 0 (Reference) | 0 (Reference) |
|  | 1 | 0.62 (0.27, 0.97) | 0.56 (0.19, 0.93) |
|  | 2 | 0.98 (0.45, 1.51) | 1.14 (0.63, 1.65) |
|  | 3 | 0.63 (-0.04, 1.30) | 1.33 (0.65, 2.01) |

^a^The saturated model includes dummy variables for all possible combinations of the timing of IPV (“none” is the reference category).

^b^The three critical period models include a dummy variable for IPV: 1) pre-conception only; 2) early childhood only; or 3) middle childhood only.

^c^The sensitive period model includes dummy variables for IPV pre-conception only, early childhood only and middle childhood only.

^d^The continuous accumulation model uses a single variable with the values of 0 to 3.

^e^The categorical accumulation model uses dummy variables for IPV once, twice or three times (0 times is the reference category)

Supplementary Table 5

*Sensitivity analysis: Model fit for structured life course models of the unadjusted association between IPV and child externalizing and internalizing behavior in boys and girls.*

|  | Log  likelihood | P  value | Scaling  correct | Free  Params |
| --- | --- | --- | --- | --- |
| **Externalizing** |  |  |  |  |
| **Boys (unadjusted)** |  |  |  |  |
| Saturated^a^ | -4798.48 | NA | 1.1654 | 10 |
| Critical: pre-conception^b^ | -4808.429 | 0.007 | 1.2197 | 4 |
| Critical: early childhood^b^ | -4809.535 | 0.005 | 1.1285 | 4 |
| Critical: middle childhood^b^ | -4807.909 | 0.010 | 1.2174 | 4 |
| Sensitive period^c^ | -4806.414 | 0.009 | 1.1568 | 6 |
| Accumulation: Continuous^d^ | **-4800.626** | **0.715** | **1.1788** | **4** |
| Accumulation: Categorical^e^ | -4800.23 | 0.540 | 1.1899 | 6 |
| Empty model | -4809.665 | 0.006 | 1.2215 | 3 |
| **Girls (unadjusted)** |  |  |  |  |
| Saturated^a^ | -4316.16 | NA | 1.3194 | 10 |
| Critical: pre-conception^b^ | -4329.248 | 0.003 | 1.3133 | 4 |
| Critical: early childhood^b^ | -4330.811 | 0.002 | 1.2173 | 4 |
| Critical: middle childhood^b^ | -4330.23 | 0.002 | 1.2785 | 4 |
| Sensitive period^c^ | -4328.176 | 0.003 | 1.1805 | 6 |
| Accumulation: Continuous^d^ | **-4319.526** | **0.527** | **1.3331** | **4** |
| Accumulation: Categorical^e^ | -4318.818 | 0.402 | 1.3195 | 6 |
| Empty model | -4331.122 | 0.002 | 1.3602 | 3 |
| **Internalizing** |  |  |  |  |
| **Boys (unadjusted)** |  |  |  |  |
| Saturated^a^ | -4340.05 | NA | 1.425 | 10 |
| Critical: pre-conception^b^ | -4357.17 | <.001 | 1.5575 | 4 |
| Critical: early childhood^b^ | -4357.18 | <.001 | 1.5660 | 4 |
| Critical: middle childhood^b^ | -4349.49 | 0.019 | 1.6922 | 4 |
| Sensitive period^c^ | -4349.10 | 0.012 | 1.4394 | 6 |
| Accumulation: Continuous^d^ | -4347.56 | 0.071 | 1.6231 | 4 |
| Accumulation: Categorical^e^ | **-4344.69** | **0.140** | **1.4816** | **6** |
| Empty model | -4357.20 | <.001 | 1.7680 | 3 |
| **Girls (unadjusted)** |  |  |  |  |
| Saturated^a^ | -4008.07 | NA | 1.4267 | 10 |
| Critical: pre-conception^b^ | -4027.144 | <.001 | 1.9185 | 4 |
| Critical: early childhood^b^ | -4033.366 | <.001 | 1.7656 | 4 |
| Critical: middle childhood^b^ | -4033.145 | <.001 | 1.7498 | 4 |
| Sensitive period^c^ | -4027.043 | <.001 | 1.5347 | 6 |
| Accumulation: Continuous^d^ | **-4013.665** | **0.133** | **1.8551** | **4** |
| Accumulation: Categorical^e^ | -4013.284 | 0.032 | 1.7180 | 6 |
| Empty model | -4033.397 | <.001 | 2.0899 | 3 |

^a^The saturated model includes dummy variables for all possible combinations of the timing of IPV (“none” is the reference category).

^b^The three critical period models include a dummy variable for IPV: 1) pre-conception only; 2) early childhood only; or 3) middle childhood only.

^c^The sensitive period model includes dummy variables for IPV pre-conception only, early childhood only and middle childhood only.

^d^The continuous accumulation model uses a single variable with the values of 0 to 3.

^e^The categorical accumulation model uses dummy variables for IPV once, twice or three times (0 times is the reference category)

Supplementary Table 6

*Sensitivity analysis: Model fit for structured life course models of the adjusted association between IPV and child externalizing and internalizing behavior in boys and girls.*

|  | Log  likelihood | P  value | Scaling  correct | Free  Params |
| --- | --- | --- | --- | --- |
| **Externalizing** |  |  |  |  |
| **Boys (adjusted)** |  |  |  |  |
| Saturated^a^ | -4753.47 | NA | 1.1276 | 14 |
| Critical: pre-conception^b^ | -4762.202 | 0.017 | 1.1268 | 8 |
| Critical: early childhood^b^ | -4763.137 | 0.012 | 1.0862 | 8 |
| Critical: middle childhood^b^ | -4761.444 | 0.028 | 1.1278 | 8 |
| Sensitive period^c^ | -4760.248 | 0.021 | 1.1090 | 10 |
| Accumulation: Continuous^d^ | **-4755.901** | **0.647** | **1.1095** | **8** |
| Accumulation: Categorical^e^ | -4755.329 | 0.507 | 1.1287 | 10 |
| Empty model | -4763.165 | 0.017 | 1.1178 | 7 |
| **Girls (adjusted)** |  |  |  |  |
| Saturated^a^ | -4239.66 | NA | 1.2188 | 14 |
| Critical: pre-conception^b^ | -4252.129 | 0.005 | 1.1538 | 8 |
| Critical: early childhood^b^ | -4254.145 | 0.002 | 1.0957 | 8 |
| Critical: middle childhood^b^ | -4253.981 | 0.001 | 1.1423 | 8 |
| Sensitive period^c^ | -4250.298 | 0.008 | 1.0923 | 10 |
| Accumulation: Continuous^d^ | **-4243.858** | **0.369** | **1.1671** | **8** |
| Accumulation: Categorical^e^ | -4243.207 | 0.236 | 1.1950 | 10 |
| Empty model | -4255.035 | 0.001 | 1.1594 | 7 |
| **Internalizing** |  |  |  |  |
| **Boys (adjusted)** |  |  |  |  |
| Saturated^a^ | -4312.55 | NA | 1.3173 | 14 |
| Critical: pre-conception^b^ | -4324.994 | 0.005 | 1.2979 | 8 |
| Critical: early childhood^b^ | -4325.004 | 0.005 | 1.3014 | 8 |
| Critical: middle childhood^b^ | **-4317.781** | **0.217** | **1.3610** | **8** |
| Sensitive period^c^ | -4317.698 | 0.127 | 1.2712 | 10 |
| Accumulation: Continuous^d^ | -4319.696 | 0.086 | 1.3372 | 8 |
| Accumulation: Categorical^e^ | -4317.651 | 0.103 | 1.3145 | 10 |
| Empty model | -4325.005 | 0.007 | 1.3544 | 7 |
| **Girls (adjusted)** |  |  |  |  |
| Saturated^a^ | -3997.53 | NA | 1.2947 | 14 |
| Critical: pre-conception^b^ | -4012.270 | <.001 | 1.4527 | 8 |
| Critical: early childhood^b^ | -4017.882 | <.001 | 1.3740 | 8 |
| Critical: middle childhood^b^ | -4017.355 | <.001 | 1.3716 | 8 |
| Sensitive period^c^ | -4011.959 | <.001 | 1.3176 | 10 |
| Accumulation: Continuous^d^ | **-4003.349** | **0.114** | **1.4162** | **8** |
| Accumulation: Categorical^e^ | -4003.045 | 0.025 | 1.4172 | 10 |
| Empty model | -4017.904 | <.001 | 1.4587 | 7 |

^a^The saturated model includes dummy variables for all possible combinations of the timing of IPV (“none” is the reference category).

^b^The three critical period models include a dummy variable for IPV: 1) pre-conception only; 2) early childhood only; or 3) middle childhood only.

^c^The sensitive period model includes dummy variables for IPV pre-conception only, early childhood only and middle childhood only.

^d^The continuous accumulation model uses a single variable with the values of 0 to 3.

^e^The categorical accumulation model uses dummy variables for IPV once, twice or three times (0 times is the reference category)

Supplementary Figure 1

*Structured life course models for the adjusted association between Intimate Partner Violence in preconception, early and middle childhood and externalizing behaviour (SDQ) in boys (n = 1903).*


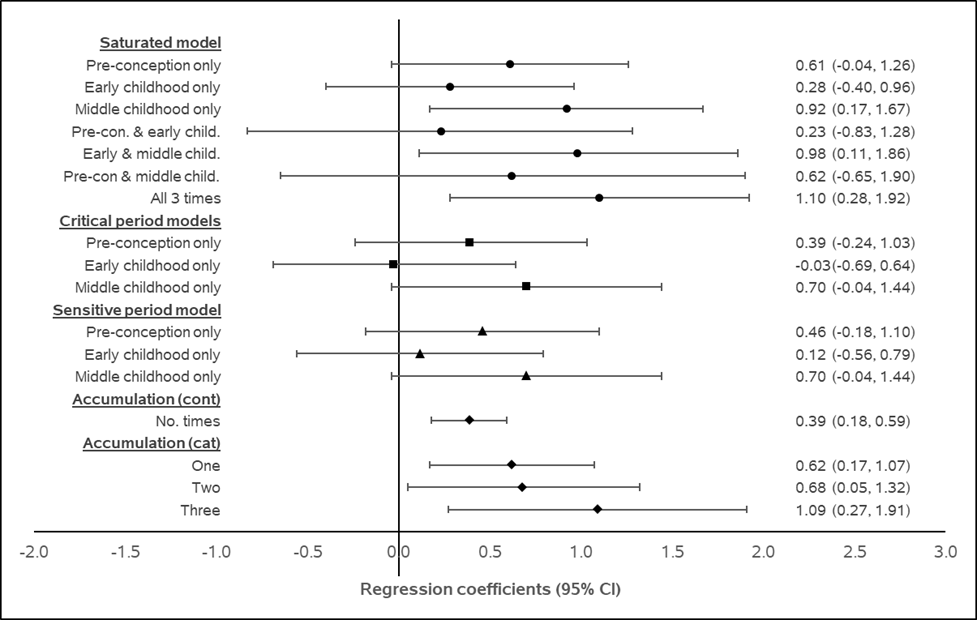


Supplementary Figure 2

*Structured life course models for the adjusted association between Intimate Partner Violence in preconception, early and middle childhood and externalizing behaviour (SDQ) in girls (n = 1794).*


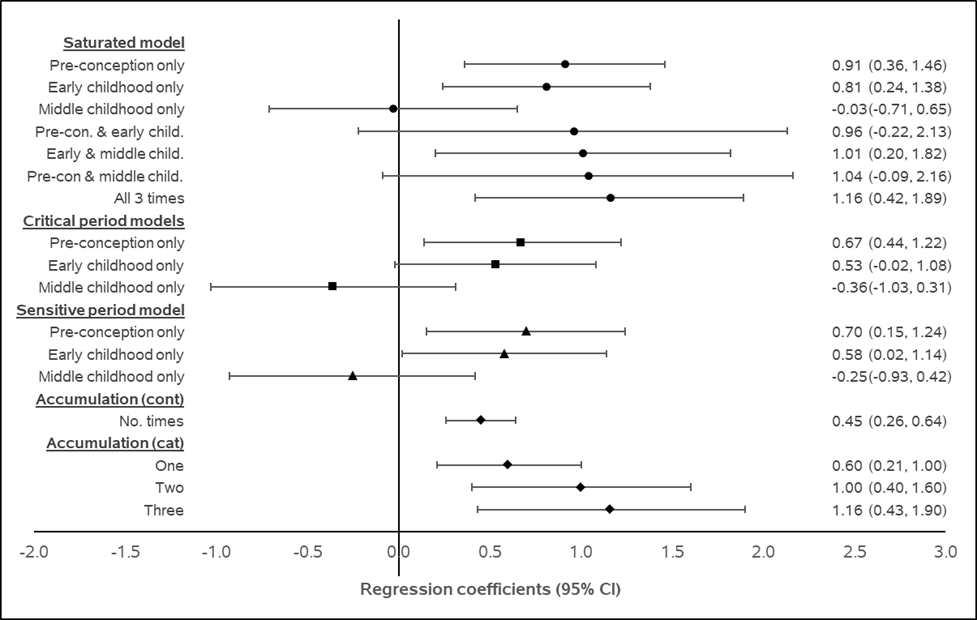


Supplementary Figure 3

*Structured life course models for the adjusted association between Intimate Partner Violence in preconception, early and middle childhood and internalizing behaviour (SDQ) in girls (n = 1794).*


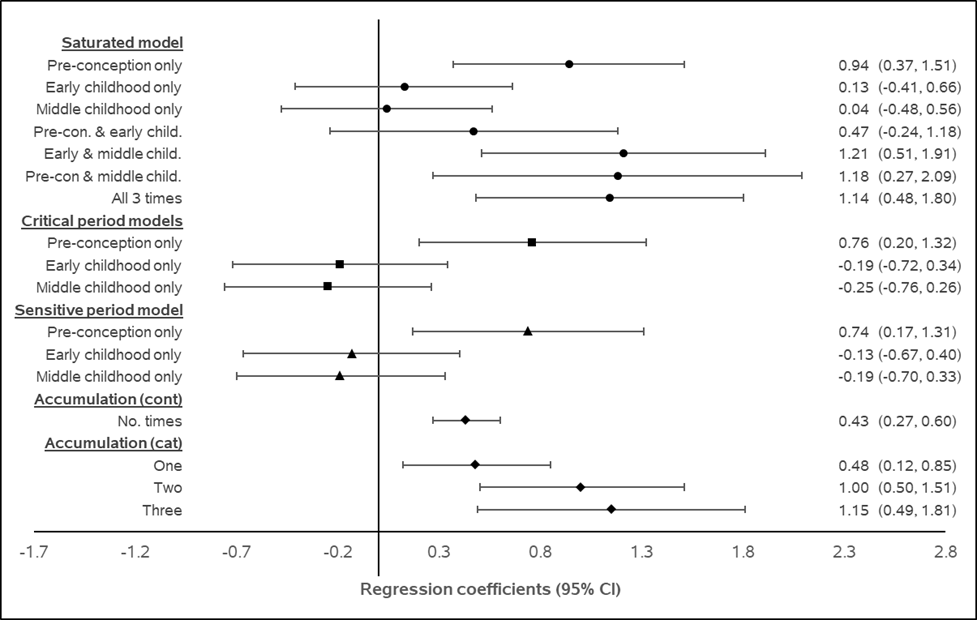


Supplementary Figure 4

*Structured life course models for the adjusted association between Intimate Partner Violence in preconception, early and middle childhood and internalizing behaviour (SDQ) in boys (n = 1903).*


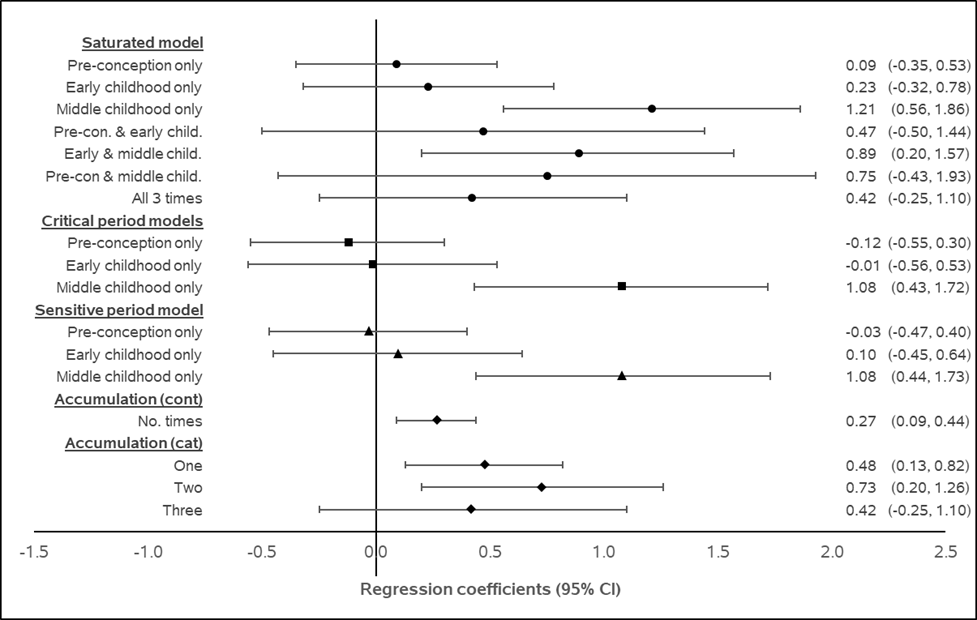

Supplement: sj-docx-1-jiv-10.1177_08862605231174505 – Supplemental material for Does Timing Matter? Associations Between Intimate Partner Violence Across the Early Life Course and Internalizing and Externalizing Behavior in Children [file sj-docx-1-jiv-10.1177_08862605231174505.docx]
